# Differential PKA activation and AKAP association determines cell fate in cancer cells

**DOI:** 10.1186/1750-2187-8-10

**Published:** 2013-10-01

**Authors:** Erik D Hedrick, Ekta Agarwal, Premila D Leiphrakpam, Katie L Haferbier, Michael G Brattain, Sanjib Chowdhury

**Affiliations:** 1 Eppley Cancer Center, University of Nebraska Medical Center, Nebraska Medical Center, Omaha, NE 68198-5950, USA

**Keywords:** Colorectal cancer, IGF1R, AKAP149, Praja2, PKA, XIAP

## Abstract

**Background:**

The dependence of malignant properties of colorectal cancer (CRC) cells on IGF1R signaling has been demonstrated and several IGF1R antagonists are currently in clinical trials. Recently, we identified a novel pathway in which cAMP independent PKA activation by TGFβ signaling resulted in the destabilization of survivin/XIAP complex leading to increased cell death. In this study, we evaluated the effect of IGF1R inhibition or activation on PKA activation and its downstream cell survival signaling mechanisms.

**Methods:**

Small molecule IGF1R kinase inhibitor OSI-906 was used to test the effect of IGF1R inhibition on PKA activation, AKAP association and its downstream cell survival signaling. In a complementary approach, ligand mediated activation of IGF1R was performed and AKAP/PKA signaling was analyzed for their downstream survival effects.

**Results:**

We demonstrate that the inhibition of IGF1R in the IGF1R-dependent CRC subset generates cell death through a novel mechanism involving TGFβ stimulated cAMP independent PKA activity that leads to disruption of cell survival by survivin/XIAP mediated inhibition of caspase activity. Importantly, ligand mediated activation of the IGF1R in CRC cells results in the generation of cAMP dependent PKA activity that functions in cell survival by inhibiting caspase activity. Therefore, this subset of CRC demonstrates 2 opposing pathways organized by 2 different AKAPs in the cytoplasm that both utilize activation of PKA in a manner that leads to different outcomes with respect to life and death. The cAMP independent PKA activation pathway is dependent upon mitochondrial AKAP149 for its apoptotic functions. In contrast, Praja2 (Pja2), an AKAP-like E3 ligase protein was identified as a key element in controlling cAMP dependent PKA activity and pro-survival signaling. Genetic manipulation of AKAP149 and Praja2 using siRNA KD had opposing effects on PKA activity and survivin/XIAP regulation.

**Conclusions:**

We had identified 2 cytoplasmic pathways dependent upon the same enzymatic activity with opposite effects on cell fate in terms of life and death. Understanding the specific mechanistic functions of IGF1R with respect to determining the PKA survival functions would have potential for impact upon the development of new therapeutic strategies by exploiting the IGF1R/cAMP-PKA survival signaling in cancer.

## Background

The IGF1R signaling pathway plays a crucial role in cell growth, proliferation, survival, and differentiation [1-5]. IGF1R is often overexpressed and upregulated in many cancer types, including colorectal cancer (CRC) [6]. Thus, IGF1R has been shown to be a promising therapeutic target and both pharmacological and biological agents have been developed to inhibit IGF1R for therapeutic applications in cancer. These agents include monoclonal antibodies, which specifically bind to IGF1R homodimers [3,7,8] and small molecular kinase inhibitors [3,7]. OSI-906 is a small molecule IGF1R kinase inhibitor that is currently in clinical trials [7]. OSI-906 targets both IR and IGF1R heterodimers [7]. This drug has been shown in previous studies to be an effective inhibitor of IGF1R signaling leading to a decrease in cellular proliferation and increased apoptosis [7]. OSI-906 has been shown to reduce tumor growth in athymic nude mice [7].

Recently, we showed that TGFβ mediates its tumor suppressor and pro-apoptotic effects in part, through the activation of protein kinase A (PKA) in a cyclic AMP (cAMP) independent manner in colorectal cancer [9]. The TGFβ mediated cAMP independent PKA activation was Smad3-dependent and inhibited the expression of the X-linked inhibitor of apoptosis protein (XIAP) that has been shown to mediate aberrant cell survival and metastasis [9,10]. Cell fate in response to cellular stress is determined by multiple signals that determine whether pro-apoptotic or anti-apoptotic signals that normally function in equilibrium will ultimately predominate in response to the stress. For example, stress causes the mitochondria to release survivin and XIAP to the cytoplasm forming a survivin/XIAP complex to promote cell survival [11]. The survivin/XIAP complex that mediates caspase inhibition has been shown to be a key cell survival mechanism enabling the metastatic process [11,12]. The complex is critical for stabilization of XIAP to inhibit caspases. We recently demonstrated that TGFβ/PKA signaling leads to the disruption and subsequent destabilization of the survivin/XIAP complex to enable cell death by PP2A mediated inhibition of Akt phosphorylation of a stabilizing XIAP site (S87) and by the direct phosphorylation of survivin at S20 which disrupts complex formation by the 2 inhibitor of apoptosis (IAP) family members and leads to their destabilization thereby enabling cell death [9,13,14].

A-kinase anchoring proteins (AKAPs) are specialized anchoring proteins that recruit and compartmentalize PKA and other enzymes in the cytoplasm to specific subcellular locations and organelles for their enzymatic functions [15,16]. This is accomplished by the binding of PKA regulatory subunits (PKARI and/or PKARII) to specific AKAPs. In our previous work, we demonstrated the critical role of AKAP149 (also termed as D-AKAP1 and AKAP121) in regulating cAMP independent TGFβ/PKA signaling for its metastasis suppressor activity [9]. Recently, Lignitto et al. (2011) described the role of Praja2 (also termed as Pja2), an AKAP-like E3 ubiquitin ligase that plays a critical role in the cAMP-dependent activation of PKA. Praja2 acts as a pro-survival AKAP and promotes cell proliferation and growth [17].

In the current work, we investigated the molecular mechanisms that determine AKAP-PKA-driven cell fate in the IGF1R-dependent subsets of CRC cells using the IGF1R kinase inhibitor OSI-906. We report a newly identified form of crosstalk between the opposing IGF1R and TGFβ signaling pathways in which PKA activation is utilized by both pathways, but different AKAPs are utilized to organize opposing functions with regard to XIAP protein stability in controlling cell survival in CRC cells. This is the first report demonstrating the novel linkage of IGF1R/cAMP-PKA/Praja2/XIAP cell survival signaling in CRC. Identification of specific mechanistic functions of IGF1R/PKA signaling could have potential for impact upon the development of therapeutic strategies for IGF1R-dependent cancer by shifting the balance of these 2 pathways away from cell survival towards cell death.

## Results

### Inhibition of IGF1R by OSI-906 activates PKA

**Figure 1:**
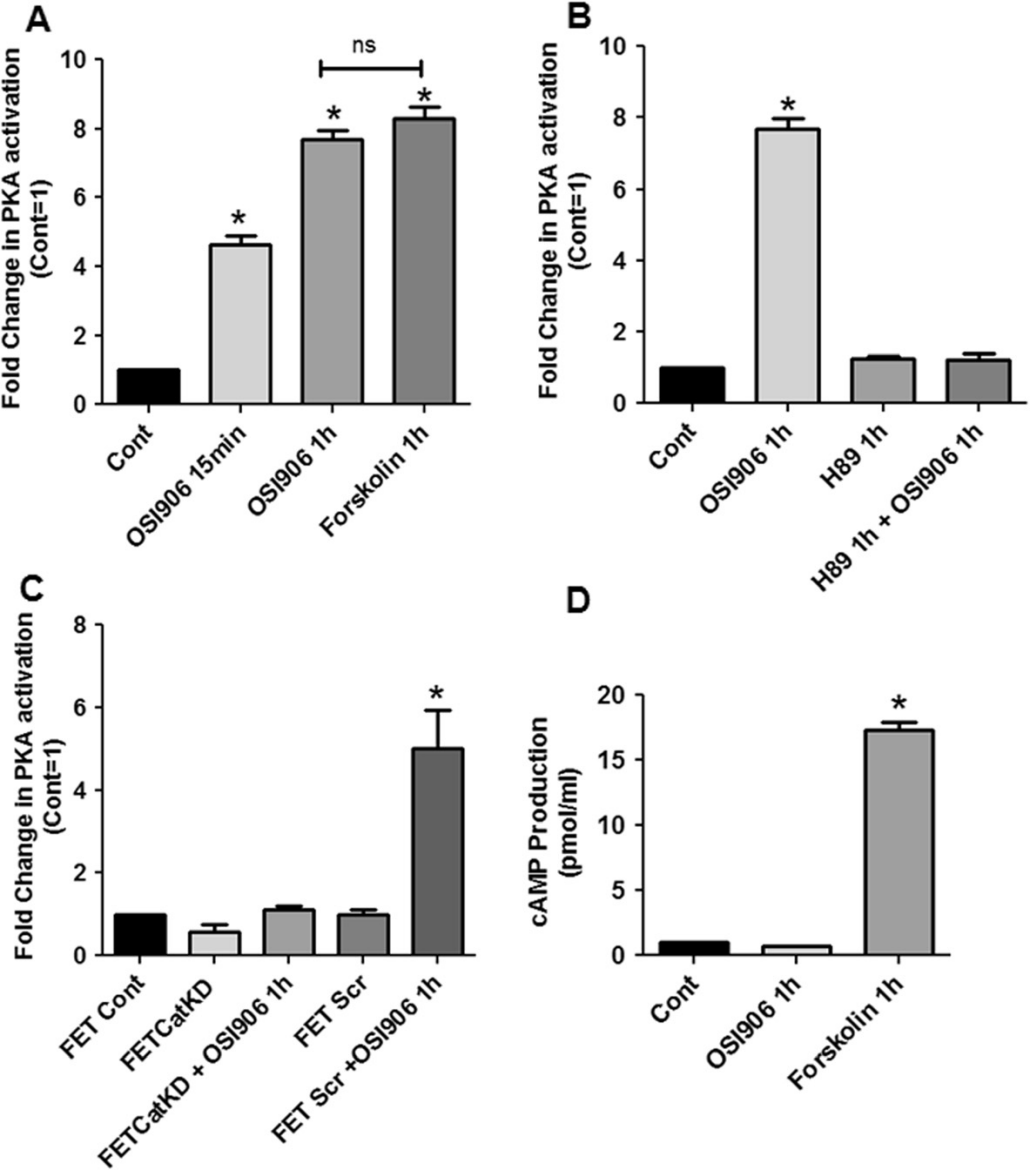
**OSI-906 mediates cAMP independent and AKAP dependent PKA activation in colon cancer cells.** FET cells treated with OSI-906 (1 μM) activated PKA in a time dependent fashion **(A)**. Forskolin (10 μM) was used as a positive control. PKA inhibitor H89 (15 μM) was used to inhibit PKA activation. Pretreatment with H89 followed by OSI-906 treatment abrogated the PKA activation **(B)**. Knockdown of PKA Catalytic subunit (FETCatKD) abrogated OSI-906 mediated PKA activation. However, FET cells transfected with Scrambled siRNA (FET Scr) cells showed PKA activation following OSI-906 treatment. **(C)**. PKA activation by OSI-906 is cAMP independent as determined via cAMP assay **(D)**. All experiments were repeated three times independently with 3 replicas per repeat for the ANOVA analysis. (* P < 0.001).

Zhang et al. (2004) first demonstrated that TGFβ could activate PKA in a Smad3-dependent manner independent of cAMP [18]. We demonstrated that TGFβ signaling can activate PKA by a Smad3-dependent, cAMP independent mechanism in IGF1R-dependent subsets of CRC cells leading to cell death through the disruption of the formation survivin/XIAP complexes which are necessary for caspase 3,7,9 inhibition to block apoptosis [9,13]. This raised the possibility that the inhibition of IGF1R in this subset might generate cell death through mechanisms involving TGFβ/PKA signaling mediated control of aberrant cell survival. Therefore, we tested the hypothesis that IGF1R inhibition by OSI-906 activates PKA in CRC cells in a cAMP independent, Smad3-dependent mechanism. To this end, IGF1R-dependent FET colon carcinoma cells [19] were treated with OSI-906 for specific times (15 mins and 1 h), and a non-radioactive protein kinase assay (Promega) was performed for measuring PKA activity. Following drug treatment, PKA activity increased by approximately 5-fold at 15 min and 7-fold by 1 h (Figure 1A). It was observed that OSI-906 mediated PKA activation was completely abolished following pretreatment of the cells with H89, a pharmacological PKA inhibitor (Figure 1B) thus indicating that PKA signaling was initiated by an endogenous cellular mechanism in response to blockade of IGF1R signaling. Similar results were confirmed in 2 additional IGF1R-dependent colon carcinoma cell lines GEO and CBS (Additional file 1: Figure S1). The OSI-906 mediated PKA activation was further confirmed by siRNA knockdown of PKA catalytic subunit. As shown in Figure 1C, knockdown of PKA catalytic subunit in FET cells (termed as FETCatKD) resulted in abrogation of OSI-906 mediated PKA activation. However, FET cells transfected with scrambled siRNA (termed FET Scr) showed PKA activation upon treatment with OSI-906.

To further confirm the activation of PKA by IGF1R inhibition, we used MK-0646, a recombinant humanized monoclonal antibody against IGF1R. Previous studies have reported that MK-0646 binds specifically to IGF1R and triggers internalization of its receptors and degradation which subsequently blocks IGF-I and IGF-II-mediated cell proliferation and survival [20]. As shown in Additional file 1: Figure S2, treatment with MK-0646 activated PKA in FET cells. We next determined whether activation of PKA by OSI-906 is dependent upon cAMP activation by treating FET cells with OSI-906 and measuring cAMP levels using a non-radioactive cAMP enzyme immunoassay (Figure 1D). It was observed that OSI-906 was unable to increase cAMP production in contrast to Forskolin treatment which provided a significant increase in cAMP levels as expected. It should be noted that the levels of OSI-906 driven PKA activity in the absence of cAMP were similar to those induced by Forskolin in its cAMP-dependent PKA activation, thus indicating that the mechanism of cAMP independent PKA activation by OSI-906 was approximately as potent as that of Forskolin induction. 1way ANOVA with Bonferroni’s multiple comparison test showed that while both treatment with OSI-906 1 h and Forskolin 1 h showed statistically significant increase in PKA activity (as indicated in Figure 1A), no statistically significant difference was observed comparing OSI-906 1 h vs. Forskolin 1 h.

### OSI-906 mediated activation of PKA and cellular apoptosis requires TGFβ signaling

**Figure 2:**
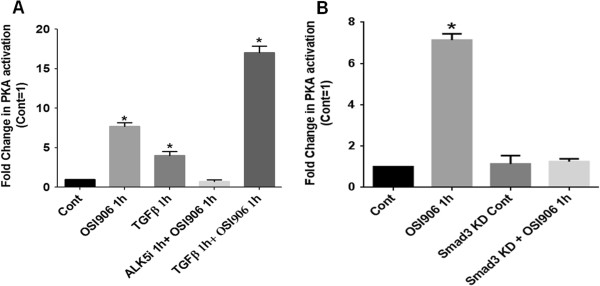
**OSI-906 activates PKA in a TGFβ signaling dependent manner.** FET cells pretreated treated with TGFβRI inhibitor ALK5i (400 nM) abrogated OSI-906 (1 μM) mediated PKA activation. FET cells treated with TGFβ (5 ng/ml) and OSI-906 together showed an increase in PKA activation **(A)**. OSI-906 mediated PKA activation is Smad3-dependent as determined by knockdown of Smad3 in FET cells **(B)**. All experiments were repeated three times independently with 3 replicas per repeat for the ANOVA analysis (* P < 0.001).

Based on cAMP independent PKA activation by OSI-906 treatment and our prior report showing TGFβ/PKA regulated aberrant cell survival, we hypothesized that OSI-906 mediated cAMP independent PKA activation requires TGFβ signaling in order to mediate its pro-apoptotic effects. To this end, FET cells were treated with either ALK5i (400 nM), an inhibitor of TGFβRI kinase activity, or exogenous TGFβ (5 ng/ml) [19]. We previously showed that pretreatment with ALK5i prior to TGFβ treatment inhibits the TGFβ/PKA-mediated cell death through the abrogation of survivin and XIAP downregulation [9,13]. This led us to the hypothesis that IGF1R inhibition leads to increased TGFβ mediated cAMP independent PKA activation which then mediates cell death. If this hypothesis were correct; blockade of TGFβ signaling would abrogate OSI-906 mediated PKA activation and downstream signaling. Figure 2A shows that pre-treatment of FET cells with ALK5i for 1 h suppressed OSI-906 mediated PKA activation thus indicating the dependence of functional TGFβ signaling for the OSI-906 effects on PKA. Treatment with TGFβ led to approximately 4-fold increase in PKA activation. However, a 16-fold increase in PKA activation was observed in cells treated with both OSI-906 and exogenous TGFβ for 1 h as shown in Figure 2A further confirming the role of TGFβ signaling in the OSI-906 effects on PKA activation. 2way ANOVA with Bonferroni’s post-tests on TGFβ- and OSI-906-mediated PKA activity demonstrated a synergistic effect on PKA activation upon combination of TGFβ and OSI-906 treatment on FET cells (p < 0.0001). Therefore, we had established that cross-talk between the IGF1R and TGFβ signaling pathways converge at PKA activation. Earlier work had emphasized the role of Smad3 in TGFβ mediated PKA activation and downstream pro-apoptotic signaling [9]. We also generated stable shRNA Smad3 knockdown in FET cells (designated FET S3KD) to genetically confirm that OSI-906 treatment was unable to activate PKA (Figure 2B).

**Figure 3:**
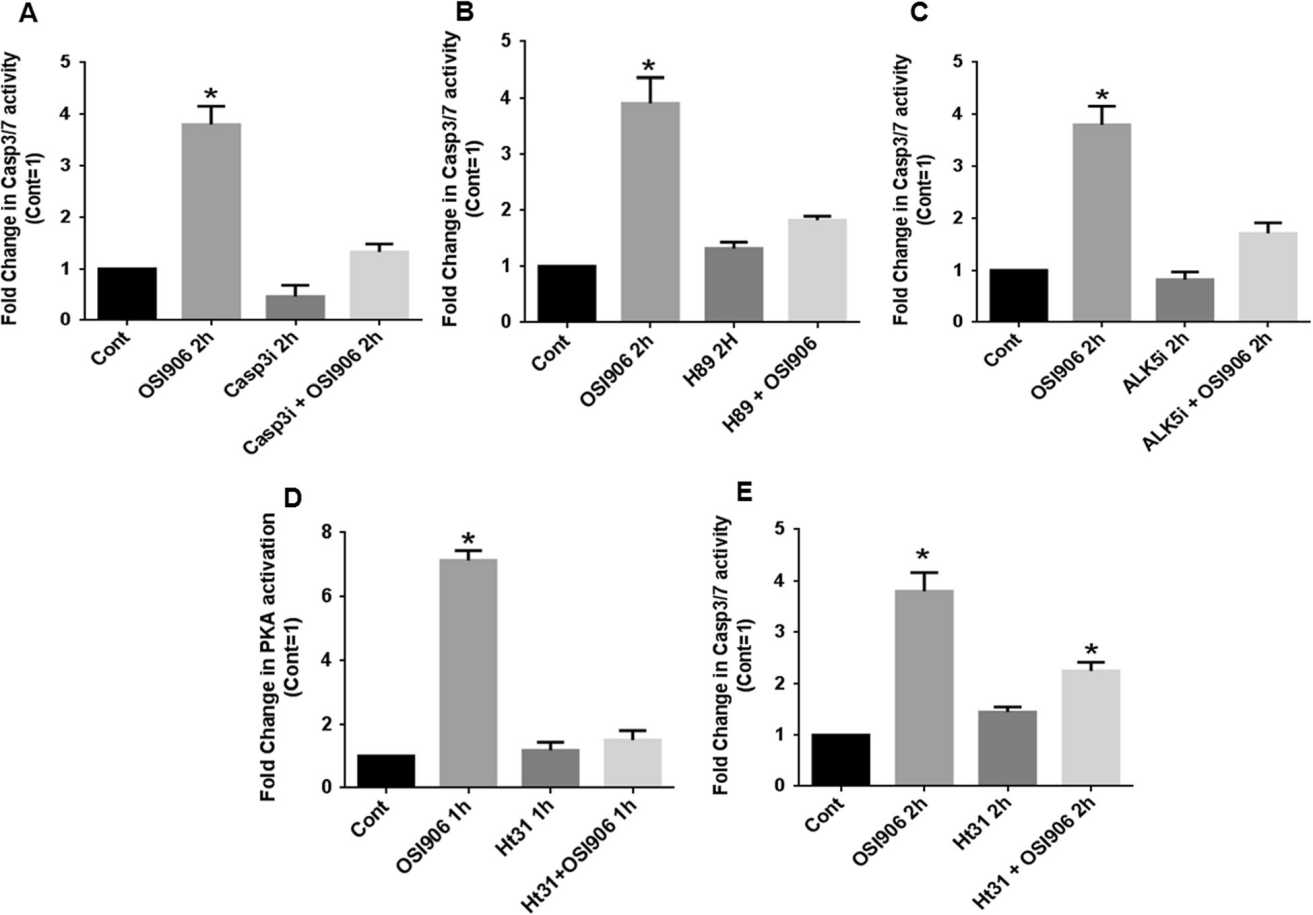
**OSI-906 mediates apoptosis by a TGFβ/PKA dependent manner.** FET cells treated with OSI-906 (1 μM) activated Caspase 3/7. Caspase 3 inhibitor C3i (20 μM) was used to inhibit Caspase 3 activation **(A)**. Caspase 3/7 activation by OSI-906 is PKA dependent as activation of Caspase 3/7 was abrogated with prior treatment of FET cells with PKA inhibitor H89 (15 μM) **(B)**. FET cells treated with TGFβRI inhibitor ALK5i (400 nM) showed abrogation of Caspase 3/7 activation. Pretreatment with ALK5i followed by OSI-906 showed similar results **(C)**. Activation of PKA by OSI-906 was abrogated in the presence of 25 μM AKAP inhibitor Ht31 **(D)**. Caspase 3/7 activation by OSI-906 is AKAP dependent as OSI-906 mediated Caspase 3/7 activation was abrogated with prior treatment of FET cells with Ht31 (25 μM) **(E)**. All experiments were repeated three times independently with 3 replicas per repeat for the ANOVA analysis (* P < 0.001).

Based on our previous report that TGFβ/PKA signaling mediated induction of cell death in CRC cells and the current finding that OSI-906 mediated PKA activation that was dependent on TGFβ signaling, we hypothesized that OSI-906 treatment would lead to TGFβ/PKA mediated cell death. We observed that OSI-906 treatment was able to increase caspase 3/7 activation by approximately 4-fold (Figure 3A). The caspase 3/7 activity was abrogated in the presence of a caspase 3 inhibitor (C3i) in both control and OSI-906 treated FET cells as expected (Figure 3A). Moreover, this caspase 3/7 activation was greatly abrogated in the presence of H89 to block PKA activity (Figure 3B), indicating that OSI-906-mediated activation of the caspase 3/7 requires the activation of PKA. The requirement for TGFβ signaling for OSI-906/PKA mediated caspase 3/7 induction was observed in FET cells in the presence of ALK5i for 1 h followed by OSI-906 treatment for 4 h. Caspase 3/7 activation was greatly abrogated as compared to FET cells treated with OSI-906 (Figure 3C). We also observed PARP cleavage associated with OSI-906 treatment. However, pre-treatment with Alk5i followed by OSI-906 treatment abrogated PARP cleavage (Additional file 1: Figure S3).

### OSI-906 mediated PKA activation and promotion of apoptosis requires AKAP

AKAPs mediate cytoskeleton dynamics, apoptosis, proliferation, growth, and other cellular processes [16]. Along with serving as anchors for PKA, AKAPs also serve as molecular scaffolds for substrates of PKA as well as other molecules and enzymes involved in specific PKA mediated biological processes [16]. Previously we showed that AKAPs are a critical component in TGFβ/PKA mediated cell survival regulation [9]. AKAP inhibitor Ht31, a synthetic thyroid anchoring peptide has been shown to be a very potent competitive inhibitor of PKARII/AKAP interaction, thereby preventing PKA anchoring. We performed PKA activity assays on FET cells to determine the extent of PKA activation following pretreatment with Ht31 (25 μM) prior to OSI-906 exposure (Figure 3D). In agreement with our earlier studies, Ht31 was able to block the OSI-906 mediated PKA activation. Since AKAP/PKA interaction appears to be a requirement for OSI-906 mediated PKA activation, we reasoned that these interactions should also be required for downstream signaling events leading to caspase 3/7 activation (Figure 3E). Addition of Ht31 inhibitor for 1 h prior to OSI-906 treatment for specified times partially abrogated the caspase 3/7 activity.

### OSI-906 inhibits the expression of XIAP in a TGFβ/PKA dependent manner

**Figure 4:**
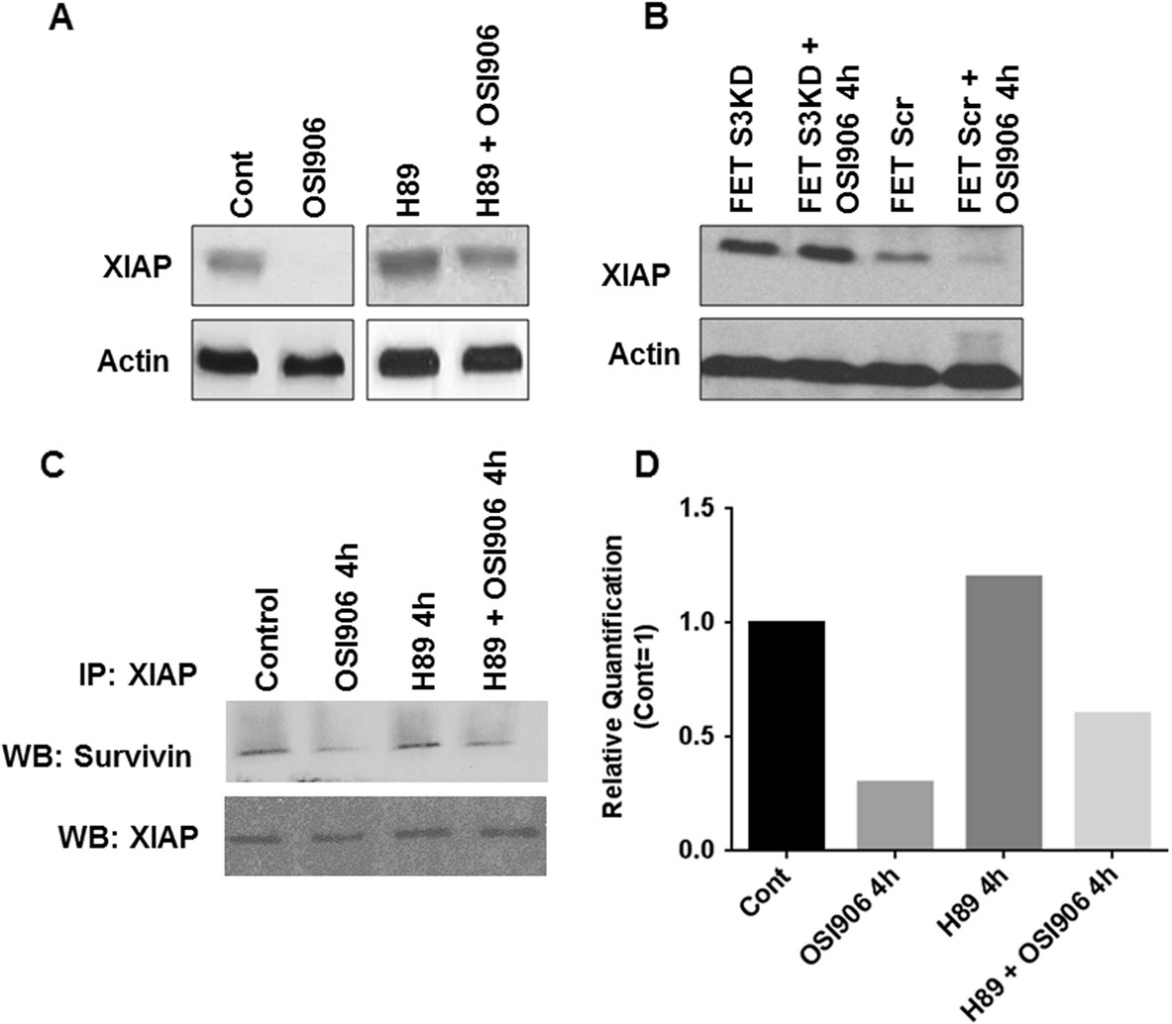
**OSI-906 regulates XIAP in a TGFβ/PKA dependent manner.** FET cells treated with OSI-906 (1 μM) showed effective downregulation of XIAP. This observed downregulation is PKA dependent as downregulation of XIAP was abrogated in FET cells treated with PKA inhibitor H89 (15 μM) **(A)**. OSI-906 mediated XIAP downregulation is dependent on Smad3 as determined by shRNA knockdown of Smad3 in FET cells (termed as FETS3KD). FETS3KD cells treated with OSI-906 showed no change in XIAP expression. However, FET Scr cells showed XIAP downregulation following treatment with OSI-906 **(B)**. FET cells treated with OSI-906 effectively dissociated XIAP from Survivin as determined by immunoprecipitation (IP) assay **(C)**. OSI-906 mediated XIAP/Survivin dissociation is PKA dependent as dissociation was abrogated when FET cells were treated with H89 and/or pretreated with H89 prior to OSI-906 treatment. Relative quantification of the IP has been performed showing reduced interaction between survivin/XIAP following OSI-906 treatments **(D)**.

XIAP plays a critical role in inhibiting apoptosis by binding to caspase 3, 7 and 9 and thereby inhibiting their pro-apoptotic activity. XIAP and survivin form a stabilizing complex that plays an important role in cell survival and metastasis [21,22]. We have reported that TGFβ/PKA signaling converges on XIAP downregulation in mediating its pro-apoptotic effects in FET cells [9]. Furthermore, it has been demonstrated that PKA activation leads to phosphorylation of survivin on Ser20 in the cytosol. This phosphorylation of survivin has been shown to disrupt survivin/XIAP binding leading to XIAP degradation [11]. We hypothesized that OSI-906 mediated PKA activation would lead to a TGFβ dependent XIAP degradation. It was observed that OSI-906 treatment led to the inhibition of XIAP expression (Figure 4A). Moreover, the OSI-906 mediated downregulation was dependent upon PKA activation as well as TGFβ signaling. When FET cells were pre-treated with H89 for 1 h prior to OSI-906 treatment, the downregulation of XIAP was abrogated and XIAP levels were stabilized (Figure 4A). To illustrate the necessity of TGFβ signaling in the OSI-906 mediated XIAP loss, stable shRNA Smad3 knockdown cell lines (FETS3KD) were utilized as described above. It was observed that OSI-906 treatment of FETS3KD cells had no effect on XIAP protein expression (Figure 4B). Furthermore, inhibition of IGF1R led to abrogation of the survivin/XIAP complex as determined by immunoprecipitation (IP) assay (Figure 4C). However, pre-treatment with H89 prevented survivin/XIAP abrogation by OSI-906. The western blots were quantified using the NIH Image J software to demonstrate the quantitative difference in survivin protein that was immunoprecipitated with XIAP antibody following OSI-906 treatment (Figure 4D). Most studies involving survivin/XIAP complex formation have been demonstrated using overexpression methods as only a small fraction of the total cellular survivin and XIAP form a complex. In our study, we used the endogenous protein as opposed to overexpression approaches to eliminate any artifacts in the interaction that may arise due to overexpression of survivin or XIAP in the parental cells. Earlier work from our laboratory has demonstrated that TGFβ treatment causes the dissociation of survivin/XIAP complex through the TGFβ/PKA signaling pathway in poorly metastatic CRC cells with functional TGFβ tumor suppressor signaling (designated CBSRII cells) as opposed to their highly metastatic counterparts deficient in functional TGFβ tumor suppressor signaling (designated CBS cells) [9].

### Differential activation of PKA by modulation of IGF1R signaling determines cell fate

**Figure 5:**
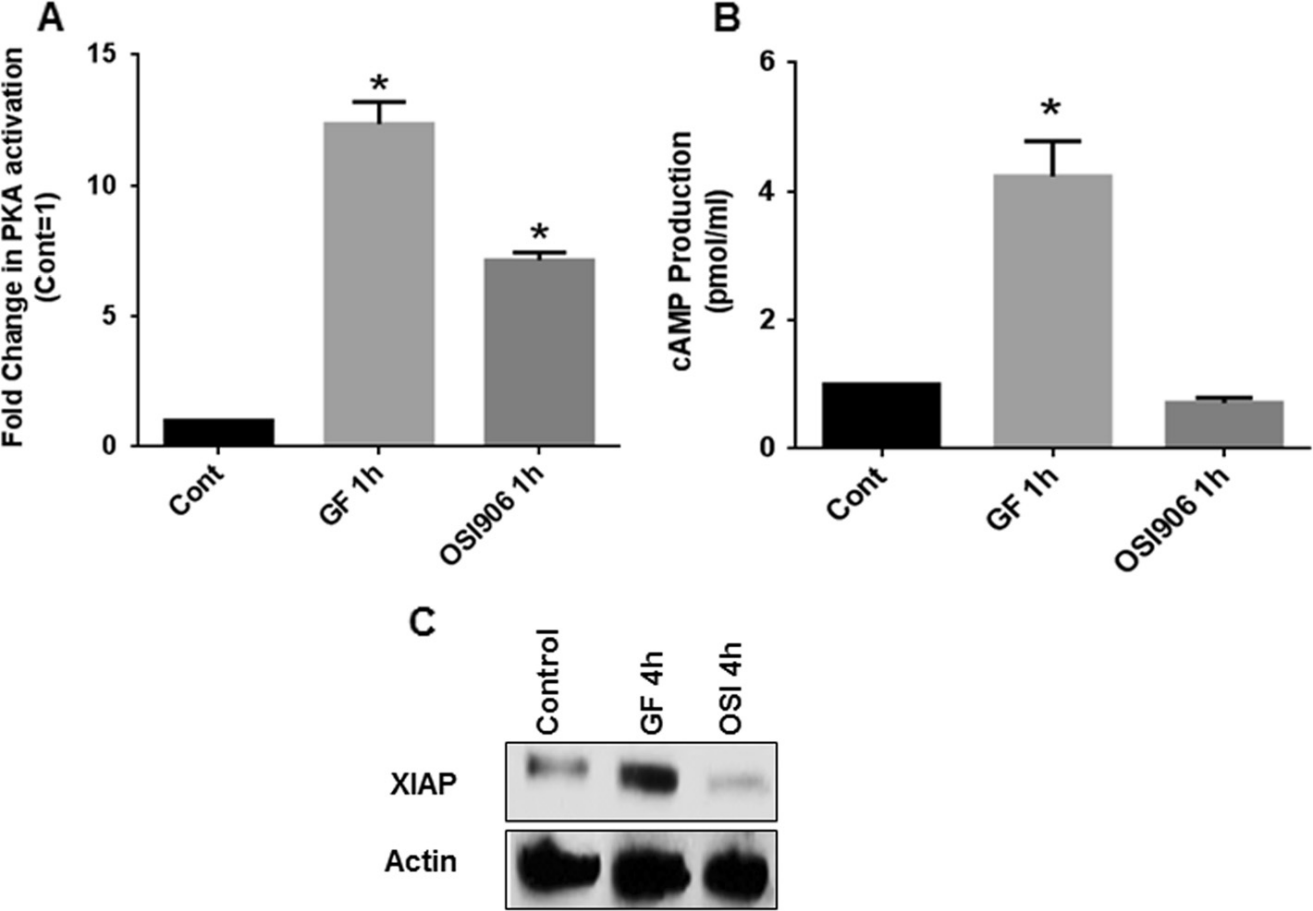
**Differential PKA activation decides cell survival or apoptotic fate.** FET cells treated with Growth Factors (or GF that includes a combination of Transferrin and Insulin) or OSI-906 (1 μM) activated PKA **(A)**. FET cells treated with GF showed cAMP-dependent activation of PKA whereas FET cells treated with OSI-906 showed cAMP-independent activation of PKA as determined by cAMP assay **(B)**. FET cells treated with GF showed an upregulation of XIAP, whereas cells treated with OSI-906 showed a downregulation of XIAP **(C)**. All experiments were repeated three times independently with 3 replicas per repeat for the ANOVA analysis (* P < 0.001).

As demonstrated above, OSI-906 mediates PKA activation in a cAMP independent, TGFβ/Smad3/AKAP149 dependent manner, leading to increased apoptosis. It is well known that classical cAMP-dependent PKA activation leads to increased cell survival signaling by transcriptional upregulation of several oncogenes. We hypothesized that ligand mediated activation of IGF1R signaling activates PKA in a cAMP-dependent manner and would lead to increased cell survival. Previous work from our laboratory have extensively used Transferrin and Insulin (jointly termed here as GF or growth factors) in combination to activate IGF-1R-mediated cell survival signaling in colon cancer cells [23-27]. Wang et al has shown that the mitogenicity of insulin (at the concentration used) in cultures of these cells is not through insulin receptor but instead through the IGF1R [27]. Intriguingly, GF mediated activation of IGF1R lead to a robust 10-fold activation of PKA (Figure 5A). This PKA activation by IGF1R was found to be cAMP-dependent as determined by cAMP assay (Figure 5B). We further observed an increased expression of XIAP following IGF1R activation (Figure 5C). This is in direct contrast to the OSI-906 mediated cAMP independent PKA activation and XIAP downregulation. Thus, the dichotomy of PKA activation decides the downstream survival or apoptotic cellular signaling fate in these subsets of CRC cells.

### Different AKAPs compete to bind PKA based on PKA activation

**Figure 6:**
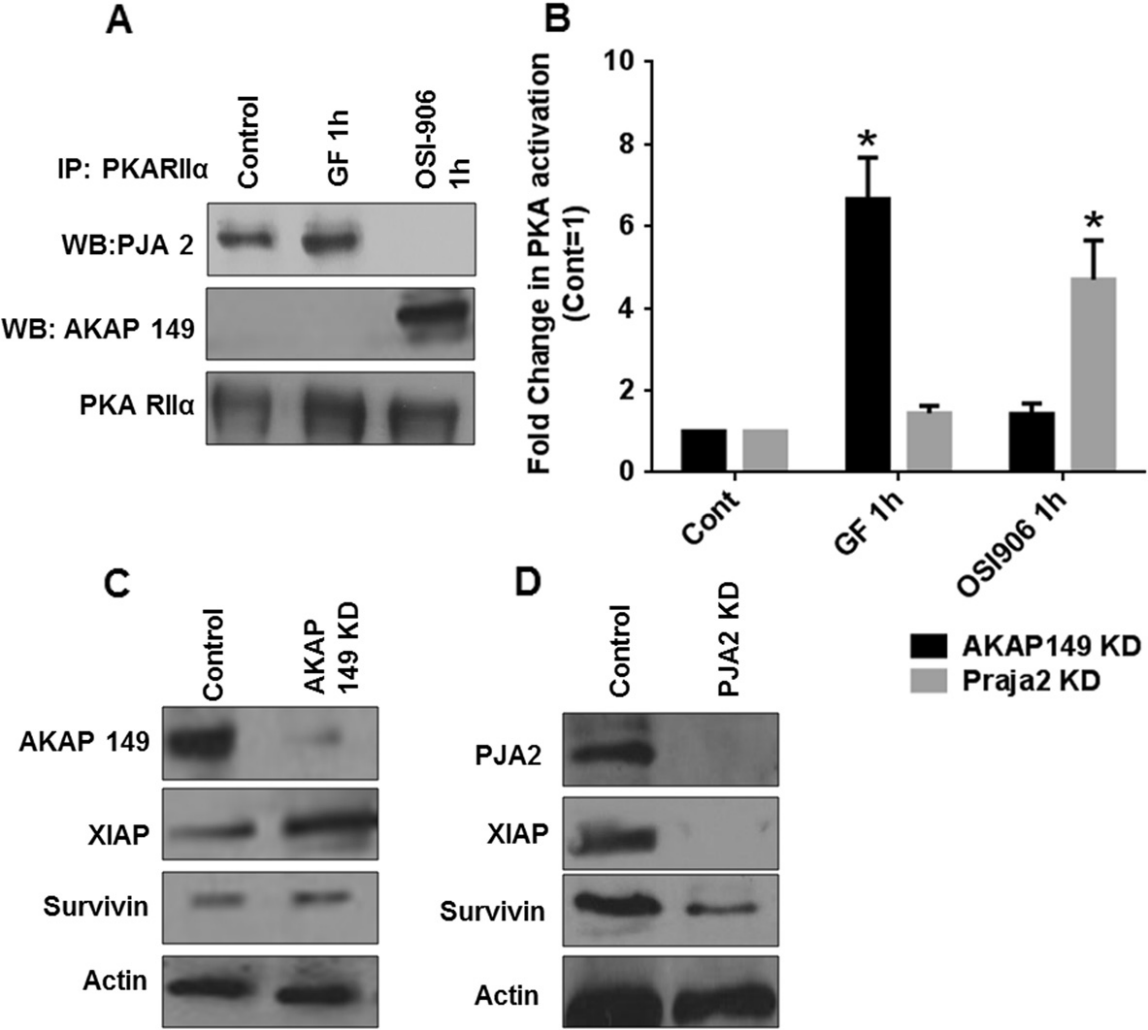
**Different AKAPs compete to bind PKA based on cAMP-dependent or independent mode of PKA activation.** FET cells treated with GF showed an effective increase in the association of Praja2 and RII subunit of PKA (PKARIIα) and a decrease in PKARII/Praja2 association when FET cells were treated with OSI-906. FET cells treated with OSI-906 showed an effective increase of AKAP149and PKARIIα association whereas an effective decrease in this association was observed when cells were treated with GF **(A)**. OSI-906-mediated PKA activation requires AKAP149association with PKA as determined by knockdown of AKAP149in FET cells. PKA activation by GF treatment requires Praja2 association with PKA as determined by knockdown of Praja2 in FET cells **(B)**. AKAP149is associated with negative regulation of IAPs survivin and XIAP as determined by knockdown of AKAP149in FET cells **(C)**. Praja2 is associated with positive regulation of survivin and XIAP as determined by knockdown of Praja2 in FET cells **(D)**. All experiments were repeated three times independently with 3 replicas per repeat for the ANOVA analysis (* P < 0.001).

Earlier work from our laboratory demonstrated the role of mitochondrial AKAP149 as playing a key role in regulating the TGFβ/PKA effects on cell survival by regulating XIAP mediated cell survival and inducing cell death in CRC cells [9]. This is critical since it is mitochondrial XIAP and survivin that move to the cytoplasm following stress response [11]. AKAP149 siRNA knockdown prevented TGFβ mediated PKA activation and XIAP downregulation [9]. Recently, Lignitto et al. [17] identified Praja2 as an AKAP-like E3 ligase scaffold which associates with the PKARII subunit in the presence of elevated concentrations of cAMP. Praja2 increases PKA activation by liberating the catalytic subunits from the regulatory subunits in a cAMP-dependent fashion. It was further demonstrated that Praja2 binds to AKAP149 [17]. We hypothesized that Praja2 recruits PKA and facilitates pro-survival functions following ligand mediated cAMP-dependent IGF1R/PKA activation. We observed that Praja2 required ligand mediated activation of IGF1R/PKA signaling to associate with the PKA RII subunit by IP analysis. However, in the presence of OSI-906, the association between Praja2 and PKA RII is completely abrogated in FET cells (Figure 6A). As demonstrated above, both activation and inhibition of IGF1R lead to a robust activation of PKA either by cAMP-dependent or independent mechanisms, respectively. Importantly, the 2 opposing mechanisms of PKA activation by OSI-906 and ligand-mediated IGF1R activation selectively bind to different AKAPs (AKAP149 or Praja2) (Figure 6A). Treatment of FET cells with OSI-906 for 1 h lead to a robust binding of PKA RII with AKAP149. However, unlike Praja2 association with PKA RII under cAMP-dependent conditions, no association of PKA RII and AKAP149 was observed under GF treatment (Figure 6A). Interestingly, robust increase in AKAP149/PKA RII association was observed under treatment with TGFβ (Additional file 1: Figure S4). These results implicate AKAP149 as a pro-apoptotic AKAP, whereas Praja2 as a pro-survival AKAP that promotes aberrant cell survival in CRC cells.

We further hypothesized that the elimination of Praja2 using siRNA knockdown would lead to increased pro-apoptotic signaling. To test this hypothesis, we transiently knocked down Praja2 and AKAP149 proteins, respectively, by siRNA in FET cells. As shown in Figure 6B, siRNA knockdown of AKAP149 completely abrogated OSI-906 mediated PKA activation. However, ligand-mediated activation of IGF1R led to robust PKA activation in the AKAP149 knockdown cells. Interestingly, Praja2 siRNA knockdown showed the opposite results with an increase in OSI-906 mediated PKA activation (Figure 6B). Previously we showed that AKAP149 siRNA knockdown led to an increase in XIAP mediated cell survival and inhibition of apoptosis [9]. As shown in Figures 6C and 6D, siRNA knockdown of AKAP149 leads to increased survivin and XIAP expression whereas transient knockdown of Praja2 leads to a decrease in survivin and XIAP expression. These data further confirms the role of AKAP149 and Praja2 in pro-apoptotic and pro-survival signaling, respectively.

## Discussion

We have shown that IGF1R and TGFβ engage in a previously unidentified form of crosstalk that controls cell survival by opposing modes of PKA activation with respect to dependence upon cAMP. The IGF1R kinase inhibitor OSI-906 was associated with TGFβ mediated PKA activation that was independent of cAMP, but was dependent on AKAP as shown by the use of an AKAP inhibitor Ht31. Previous work from our laboratory has established the role of AKAP149 in TGFβ/PKA tumor suppressor signaling. In the present work, we observed that OSI-906 also mediates its pro-apoptotic effects through AKAP149, as determined by immunoprecipitation and knockdown studies (Figures 6A-6D). The OSI-906 mediated PKA activation is dependent on TGFβ signaling, as ALK5i (TGFβRI kinase inhibitor) or Smad3 KD resulted in abrogation of the OSI-906 mediated PKA activation. In addition, TGFβ treatment increased PKA activation. OSI-906 also induced caspase 3/7 mediated apoptosis and downregulation of XIAP which was dependent on TGFβ signaling and cAMP-independent PKA activation. The OSI-906 mediated downregulation of these IAPs was also abrogated in Smad3 KD cells. Survivin promotes survival by binding to the BIR3 domain of XIAP in the cytosol and prevents XIAP auto-ubiquitination, thus allowing XIAP to associate with caspases and prevent apoptosis [10,28-30]. Treatment with OSI-906 dissociated XIAP from survivin as demonstrated by co-immunoprecipitation analysis. However, this dissociation was inhibited when PKA activity was blocked by pre-treatment with H89 to abrogate PKA activity, thus illustrating the critical role of cAMP-independent PKA signaling in the OSI-906 mediated effects on apoptosis. Treatment with IGF1R-specific monoclonal activity MK-0646 also activated PKA although with a lower potency that OSI-906. This can be attributed to OSI-906 being a dual kinase IGF1R + Insulin Receptor (IR) inhibitor which might account for a more potent effect on PKA activation compared to a blocking antibody against IGF1R.

Interestingly, IGF1R activation also led to robust activation of PKA in a cAMP-dependent manner. It was determined that the IGF1R mediated PKA activation was cAMP-dependent as reflected by elevated levels of cAMP following IGF1R activation leading to a pro-survival cellular environment. An important observation from this study was the demonstration of the dependence of IGF1R mediated cAMP/PKA aberrant cell survival signaling on Praja2. Praja2 is an AKAP which is associated with the RII regulatory subunit of PKA and is dependent upon elevated cAMP levels for binding of PKA [17]. Praja2 binds to PKARII through its regulatory subunit binding domain (RBD) [17]. This AKAP-like scaffolding protein contains a RING finger domain which confers E3 ubiquitin ligase activity enabling Praja2 mediated cell survival. Lignitto et al. [17] demonstrated that in the presence of elevated cAMP levels Praja2 binds to PKARII. The binding of Praja2 with PKARII causes ubiquitination of PKARII followed by its proteasomal degradation [17]. In line with the pro-survival function of Praja2, we showed that the knockdown of Praja2 in CRC cells blocked IGF1R induced PKA activation and downregulated survivin and XIAP expression.

This study shows AKAP dependent PKA activation in the cytoplasm based on cAMP dependence or independence leads to activation of 2 opposing pathways to orchestrate cell fate in terms of life or death thus adding further levels of complexity to the many roles performed by PKA. Deregulation of AKAPs has been shown in numerous studies to lead to cancer [31]. For example, Gravin/PKA association induces cell cycle arrest and apoptosis via cyclin D1 downregulation and activation of caspase 3 respectively [31]. PKA also plays a key role in DNA replication via its association with AKAP95 [31].

**Figure 7:**
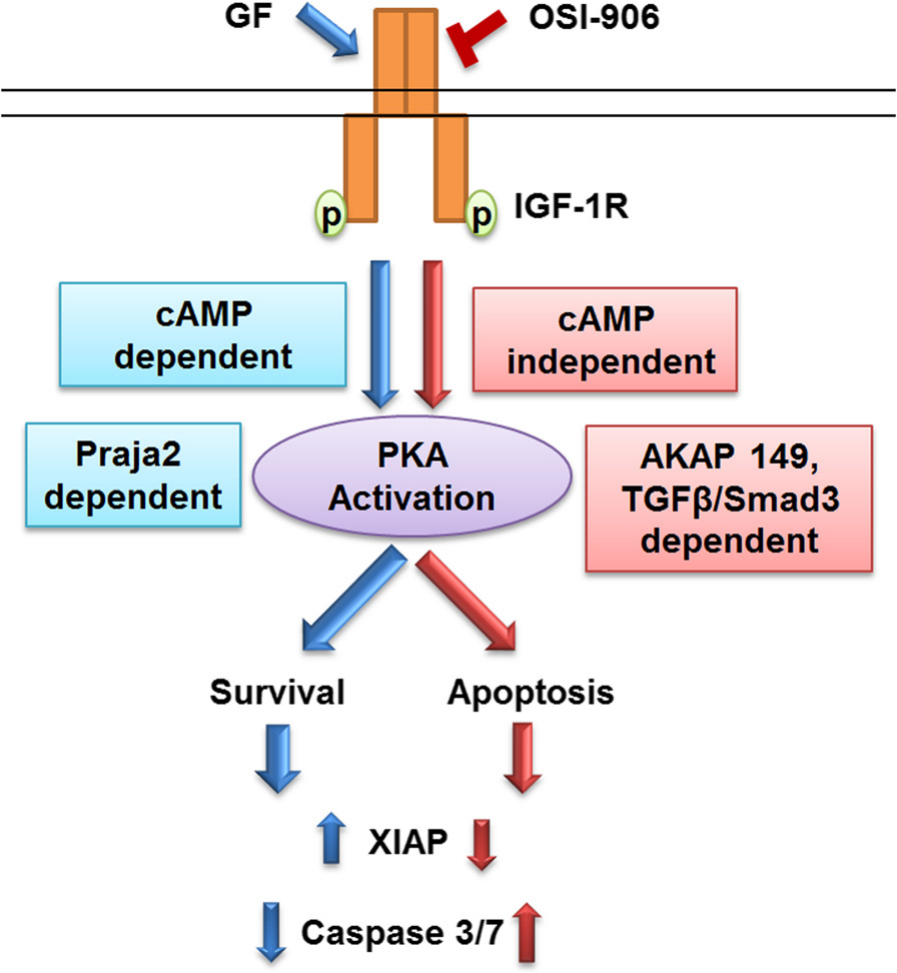
Schematic representation of AKAP/PKA signaling that regulates cell fate.

In this study, we have elucidated dual mechanisms of PKA activation. The pro-apoptotic mechanism does not require cAMP, and directs PKA to induce apoptosis in CRC cells [9]. We also show that activation of PKA in a cAMP-dependent manner via IGF1R activation resulted in PKA induced cell survival. These findings have elucidated a dichotomous role for PKA in cell survival that is ultimately determined by directing cell fate at the level of cAMP dependence and selection of binding to AKAP149 or Praja2. In essence, cAMP dependency reflects a form of novel crosstalk between TGFβ and IGF1R signaling which influences the equilibrium between cell death and cell survival. Activation of IGF1R diverts this equilibrium in favor of cell survival by activating PKA in a manner that is cAMP/ Praja2 dependent. This activation leads to the upregulation of XIAP and downregulation of caspase 3/7 activation. On the other hand, inhibition of IGF1R also leads to activation of PKA. This activation requires endogenous TGFβ signaling as well as AKAP149 association. The data indicate that the dual nature of PKA activation is accomplished by differential AKAP association. As observed, AKAP149 and Praja 2 associate with PKA in a cAMP independent or cAMP-dependent fashion respectively. This finding has been schematically represented in Figure 7. While Praja2 association leads to proteasomal degradation of PKA RII via its ubiquitin ligase activity, AKAP149 association may be enabling other molecules of the TGFβ/PKA pathway to be in close proximity with PKA to achieve specific pro-apoptotic processes. Specific molecular signatures associated with AKAP complex signaling will enhance our understanding of the functions of these AKAPs and how they might be exploited as novel targets for therapy.

## Conclusion

We have shown that IGF1R activation in cell lines of IGF1R-dependent CRC subset results in the induction of cAMP-dependent PKA activity that stimulates survivin/XIAP complex formation and mediates cell survival. Moreover, a different AKAP called Praja2 from that which generates cAMP independent activity (AKAP149) mediates this function. Thus, cellular fate with respect to life and death in response to stress in these cancer cells appears to be a cytoplasmic function that depends upon the dynamics of the interplay between these 2 AKAPs and their anchoring functions of the components of the 2 opposing pathways that they harbor. Understanding the dynamics of the molecular controls by which the cells select life or death will be a significant addition to our conceptual understanding of how AKAPs function to organize overall cytoplasmic signaling in response to changes in cellular environment. Characterization of AKAP processing of life and death signals for determining cell fate may well identify additional factors that are potential novel targets for enhancing treatment that can counter aberrant cell survival properties of cancer cells by promoting the biological consequences of cAMP independent PKA activation.

## Methods

### Cell culture and reagents

The FET, CBS and GEO colon carcinoma cells were originally isolated from CRC primary tumors and have been extensively characterized [32,33]. These cells were maintained at 37°C in humidified atmosphere in a chemically defined serum free (SF) medium containing McCoy’s 5A medium (Sigma-Aldrich) supplemented with amino acids, pyruvate, vitamins, antibiotics, and growth factors transferrin (4 μg/ml; Sigma), insulin (20 μg/ml; Sigma), and Epidermal Growth Factor (EGF) (10 ng/ml; R&D Systems). Cells were routinely subcultured with a 0.25% trypsin (Invitrogen) in Joklik’s medium (Invitrogen) containing 0.1% EDTA. Cells were harvested on day 5 after the addition of specific pharmacological agents at different time points. OSI-906 was purchased from Chemitek. TGFβ1 was purchased from R&D. MK-0646 was obtained from Merck & Co.

### Antibodies, western blotting and immunoprecipitation

Survivin antibody (#2808) and PKA inhibitor H89 (# 9844) was obtained from Cell Signaling Technology. The PKA RII (# ab124400), XIAP (#s ab21278; ab28151), Praja2 (#s ab131118; ab56876), PARP (# ab4830) and Smad3 (# ab28379) antibodies were purchased from Abcam. Anti-Actin antibody was purchased from Sigma (# A2066). The AKAP inhibitor Ht31 was obtained from Promega (# V8211). Cells were lysed in TNESV lysis buffer (50 mmol/liter Tris (pH 7.5), 150 mmol/liter NaCl, 1% NP40, 50 mmol/liter NaF, 1 mmol/liter Na3VO4, 25 μg/ml β-glycerophosphate, 1 mmol/liter phenylmethylsulfonyl fluoride (PMSF), and a protease inhibitor cocktail (Roche, Indianapolis, IN) as previously described [9,13,34,35]. Western blotting was performed using previous established protocol from our laboratory [9,13,34-36]. Co- Immunoprecipitation (IP) was performed with 500 μg protein aliquots using magnetic beads (Millipore) according to the instructions provided by the manufacturer (# LSKMAGAG10) and previously established method [9]. Binding of survivin to XIAP was tested by IP with an antibody to XIAP (Clone 2 F1) at a concentration of 2 μg/mL [37] which was selected as the optimal concentration of IP based on antibody titration and immunoblotted from survivin.

### RNA interference studies

PKAα Cat (sc-36240), Praja2 (sc-91836) and AKAP149 (sc-136355) siRNAs and Smad3 shRNA (sc-77326-SH) were obtained from Santa Cruz Biotechnology. Transfection was performed according to manufacturer’s protocol and previously established methods in our laboratory [9].

### PKA activity assay

PKA activity was measured using the PepTag non-radioactive protein kinase assay (Promega, catalogue no. V5340) using kemptide (LRRASLG) adhering to the protocol provided by the manufacturer [9]. This assay utilizes brightly colored, fluorescent peptide substrate as mentioned above, that is highly specific to PKA.

### cAMP assay

For quantitative determination of cAMP, a non-radioactive Direct Cyclic AMP Enzyme Immunoassay kit (Enzo Life Sciences, catalogue no. ADI-901-066) was employed. The protocol provided by the manufacturer was used [9].

### Caspase activity assay

Caspase 3/7 activity was measured using the Apo-ONE Homogenous Caspase 3/7 assay (Promega, catalogue no. G7790) using caspase inhibitor Ac-DEVD-CHO (Promega, catalogue no. G5961). The Apo-ONE Homogeneous Caspase 3/7 kit contains the caspase 3/7 substrate rhodamine; Z-DEVD-R110 as a profluorescent substrate. Following mixture of buffer and substrate along with cell lysates under different treatment conditions cleavage of DEVD peptides occur by caspase 3/7 activity which is measured by fluorometric methods.

### Statistical analysis

Results are expressed as mean ± SEM. Differences between groups were tested for statistical significance by using one-way Anova using GraphPad Prism 5 software. Only P values of <0.001 were considered significant. All experiments have been repeated three times independently with 3 replicas per repeat for the ANOVA analysis.

## Abbreviations

CRC: Colorectal cancer; IGF1R: Insulin-like growth factor receptor 1; AKAP: A-kinase anchoring protein; PKA: Protein kinase A; XIAP: X-linked inhibitor of apoptosis.

## Competing interests

The authors declare that they have no competing interests.

## Authors’ contributions

EDH carried out the different PKA, cAMP and Caspase assays in the study, analyzed the data and drafted the manuscript. EA, PDL, KHL helped with performing western blotting, drug treatments and transient transfection using siRNA. MGB and SC participated in the conception and design of the study and helped to draft the final manuscript. All authors read and approved the final manuscript.

## Supplementary Material

Additional file 1: Figure S1 PKA activity assay in IGF1R-dependent GEO and CBS cells. Treatment with OSI-906 (1 μM) leads to increase in PKA activation. Forskolin was used as a positive control. **Figure S2**: MK-0646 activates PKA in FET Cells. Treatment with MK-0646, a humanized recombinant monoclonal antibody against IGF-1R activates PKA FET cells. Forskolin was used as a positive control. **Figure S3**: PARP activation by OSI-906 treatment is dependent on TGFβ/PKA signaling. Treatment with OSI906 (1 μM) for 4 h lead to PARP cleavage. Pretreatment with ALK5i (400nM) or H89 (10 μM) followed by OSI-906 treatment abrogate the PARP cleavage. **Figure S4**: TGFβ Signaling regulates AKAP 149/ PKARIIα interaction in FET cells. Treatment with TGFβ (5 ng/mL) for 4 h lead to robust increase in AKAP149/PKA RII interaction. Pretreatment with Ht31 (25 μM) followed by TGFβ treatment partially abrogated the AKAP149/PKA RII interaction. Click here for file

## References

[B1] Rodon J DeSantos V Ferry RJ Jr Kurzrock R Early drug development of inhibitors of the insulin-like growth factor-I receptor pathway: lessons from the first clinical trials Mol Cancer Ther 2008 7 9 2575 2588 10.1158/1535-7163.MCT-08-0265 18790742 PMC3101870

[B2] Ryan PD Goss PE The emerging role of the insulin-like growth factor pathway as a therapeutic target in cancer Oncologist 2008 13 1 16 24 10.1634/theoncologist.2007-0199 18245009

[B3] Valentinis B Baserga R IGF-I receptor signalling in transformation and differentiation Mol Pathol 2001 54 3 133 137 10.1136/mp.54.3.133 11376123 PMC1187050

[B4] Buck E Gokhale PC Koujak S Brown E Eyzaguirre A Tao N Rosenfeld-Franklin M Lerner L Chiu MI Wild R Compensatory insulin receptor (IR) activation on inhibition of insulin-like growth factor-1 receptor (IGF-1R): rationale for cotargeting IGF1R and IR in cancer Mol Cancer Ther 2010 9 10 2652 2664 10.1158/1535-7163.MCT-10-0318 20924128

[B5] Godsland IF Insulin resistance and hyperinsulinaemia in the development and progression of cancer Clin Sci (Lond) 2009 118 5 315 332 10.1042/CS20090399 19922415 PMC2782313

[B6] Weber MM Fottner C Liu SB Jung MC Engelhardt D Baretton GB Overexpression of the insulin-like growth factor I receptor in human colon carcinomas Cancer 2002 95 10 2086 2095 10.1002/cncr.10945 12412161

[B7] Mulvihill MJ Cooke A Rosenfeld-Franklin M Buck E Foreman K Landfair D O’Connor M Pirritt C Sun Y Yao Y Discovery of OSI-906: a selective and orally efficacious dual inhibitor of the IGF-1 receptor and insulin receptor Future Med Chem 2009 1 6 1153 1171 10.4155/fmc.09.89 21425998

[B8] Pitts TM Tan AC Kulikowski GN Tentler JJ Brown AM Flanigan SA Leong S Coldren CD Hirsch FR Varella-Garcia M Development of an integrated genomic classifier for a novel agent in colorectal cancer: approach to individualized therapy in early development Clin Cancer Res 2010 16 12 3193 3204 10.1158/1078-0432.CCR-09-3191 20530704 PMC2889230

[B9] Chowdhury S Howell GM Rajput A Teggart CA Brattain LE Weber HR Chowdhury A Brattain MG Identification of a novel TGFbeta/PKA signaling transduceome in mediating control of cell survival and metastasis in colon cancer PLoS One 2011 6 5 e19335 10.1371/journal.pone.0019335 21559296 PMC3086924

[B10] Dubrez-Daloz L Dupoux A Cartier J IAPs: more than just inhibitors of apoptosis proteins Cell Cycle 2008 7 8 1036 1046 10.4161/cc.7.8.5783 18414036

[B11] Dohi T Xia F Altieri DC Compartmentalized phosphorylation of IAP by protein kinase A regulates cytoprotection Mol Cell 2007 27 1 17 28 10.1016/j.molcel.2007.06.004 17612487 PMC1986705

[B12] Mehrotra S Languino LR Raskett CM Mercurio AM Dohi T Altieri DC IAP regulation of metastasis Cancer Cell 2010 17 1 53 64 10.1016/j.ccr.2009.11.021 20129247 PMC2818597

[B13] Chowdhury S Howell GM Teggart CA Chowdhury A Person JJ Bowers DM Brattain MG Histone deacetylase inhibitor belinostat represses survivin expression through reactivation of transforming growth factor beta (TGFbeta) receptor II leading to cancer cell death J Biol Chem 2011 286 35 30937 30948 10.1074/jbc.M110.212035 21757750 PMC3162453

[B14] Agarwal E Brattain MG Chowdhury S Cell survival and metastasis regulation by Akt signaling in colorectal cancer Cell Signal 2013 25 8 1711 1719 10.1016/j.cellsig.2013.03.025 23603750 PMC3686084

[B15] Pidoux G Tasken K Specificity and spatial dynamics of protein kinase A signaling organized by A-kinase-anchoring proteins J Mol Endocrinol 2010 44 5 271 284 10.1677/JME-10-0010 20150326

[B16] Welch EJ Jones BW Scott JD Networking with AKAPs: context-dependent regulation of anchored enzymes Mol Interv 2010 10 2 86 97 10.1124/mi.10.2.6 20368369 PMC2895371

[B17] Lignitto L Carlucci A Sepe M Stefan E Cuomo O Nistico R Scorziello A Savoia C Garbi C Annunziato L Control of PKA stability and signalling by the RING ligase praja2 Nat Cell Biol 2011 13 4 412 422 10.1038/ncb2209 21423175

[B18] Zhang L Duan CJ Binkley C Li G Uhler MD Logsdon CD Simeone DM A transforming growth factor beta-induced Smad3/Smad4 complex directly activates protein kinase A Mol Cell Biol 2004 24 5 2169 2180 10.1128/MCB.24.5.2169-2180.2004 14966294 PMC350541

[B19] Wang J Yang L Yang J Kuropatwinski K Wang W Liu XQ Hauser J Brattain MG Transforming growth factor beta induces apoptosis through repressing the phosphoinositide 3-kinase/AKT/survivin pathway in colon cancer cells Cancer Res 2008 68 9 3152 3160 10.1158/0008-5472.CAN-07-5348 18451140

[B20] Donovan EA Kummar S Role of insulin-like growth factor-1R system in colorectal carcinogenesis Crit Rev Oncol Hematol 2008 66 2 91 98 10.1016/j.critrevonc.2007.09.003 17977741 PMC2374741

[B21] Schimmer AD Dalili S Targeting the IAP family of caspase inhibitors as an emerging therapeutic strategy Hematology Am Soc Hematol Educ Program 2005 215 219 16304383 10.1182/asheducation-2005.1.215

[B22] Schimmer AD Dalili S Batey RA Riedl SJ Targeting XIAP for the treatment of malignancy Cell Death Differ 2006 13 2 179 188 10.1038/sj.cdd.4401826 16322751

[B23] Awwad RA Sergina N Yang H Ziober B Willson JK Zborowska E Humphrey LE Fan R Ko TC Brattain MG The role of transforming growth factor alpha in determining growth factor independence Cancer Res 2003 63 15 4731 4738 12907656

[B24] Boyd DD Levine AE Brattain DE McKnight MK Brattain MG Comparison of growth requirements of two human intratumoral colon carcinoma cell lines in monolayer and soft agarose Cancer Res 1988 48 9 2469 2474 3281751

[B25] Hu YP Patil SB Panasiewicz M Li W Hauser J Humphrey LE Brattain MG Heterogeneity of receptor function in colon carcinoma cells determined by cross-talk between type I insulin-like growth factor receptor and epidermal growth factor receptor Cancer Res 2008 68 19 8004 8013 10.1158/0008-5472.CAN-08-0280 18829558 PMC4472475

[B26] Jiang D Yang H Willson JK Liang J Humphrey LE Zborowska E Wang D Foster J Fan R Brattain MG Autocrine transforming growth factor alpha provides a growth advantage to malignant cells by facilitating re-entry into the cell cycle from suboptimal growth states J Biol Chem 1998 273 47 31471 31479 10.1074/jbc.273.47.31471 9813060

[B27] Wang D Li W Jiang W Humphrey LE Howell GM Brattain MG Autocrine TGFalpha expression in the regulation of initiation of human colon carcinoma growth J Cell Physiol 1998 177 3 387 395 10.1002/(SICI)1097-4652(199812)177:3<387::AID-JCP2>3.0.CO;2-L 9808147

[B28] Andersen MH Svane IM Becker JC Straten PT The universal character of the tumor-associated antigen survivin Clin Cancer Res 2007 13 20 5991 5994 10.1158/1078-0432.CCR-07-0686 17947459

[B29] Eckelman BP Salvesen GS Scott FL Human inhibitor of apoptosis proteins: why XIAP is the black sheep of the family EMBO Rep 2006 7 10 988 994 10.1038/sj.embor.7400795 17016456 PMC1618369

[B30] Fabregat I Dysregulation of apoptosis in hepatocellular carcinoma cells World J Gastroenterol 2009 15 5 513 520 10.3748/wjg.15.513 19195051 PMC2653340

[B31] Almeida MQ Stratakis CA How does cAMP/protein kinase A signaling lead to tumors in the adrenal cortex and other tissues? Mol Cell Endocrinol 2011 336 1–2 162 168 21111774 10.1016/j.mce.2010.11.018 PMC3049838

[B32] Brattain MG Levine AE Chakrabarty S Yeoman LC Willson JK Long B Heterogeneity of human colon carcinoma Cancer Metastasis Rev 1984 3 3 177 191 10.1007/BF00048384 6437669

[B33] Brattain MG Marks ME McCombs J Finely W Brattain DE Characterization of human colon carcinoma cell lines isolated from a single primary tumour Br J Cancer 1983 47 3 373 381 10.1038/bjc.1983.56 6830688 PMC2011309

[B34] Chowdhury S Ongchin M Sharratt E Dominguez I Wang J Brattain MG Rajput A Intra-Tumoral Heterogeneity in Metastatic Potential and Survival Signaling between Iso-Clonal HCT116 and HCT116b Human Colon Carcinoma Cell Lines PLoS One 2013 8 4 e60299 10.1371/journal.pone.0060299 23560089 PMC3613369

[B35] Chowdhury S Ongchin M Wan G Sharratt E Brattain MG Rajput A Restoration of PTEN activity decreases metastases in an orthotopic model of colon cancer J Surg Res 2013 184 2 755 760 10.1016/j.jss.2013.03.035 23623571 PMC4096772

[B36] Chowdhury S Sharratt E Spernyak J Brattain MG Rajput A Anti-tumor activity of IGF1R kinase inhibitor PQIP in colon cancer Clin Exp Pharmacol 2013 S4 005

[B37] Jost PJ Grabow S Gray D McKenzie MD Nachbur U Huang DC Bouillet P Thomas HE Borner C Silke J XIAP discriminates between type I and type II FAS-induced apoptosis Nature 2009 460 7258 1035 1039 10.1038/nature08229 19626005 PMC2956120

