# Supplementary material for: Differential PKA activation and AKAP association determines cell fate in cancer cells
Source: J Mol Signal. 2013 Oct 1;8:10. doi: 10.1186/1750-2187-8-10 (PMC3853032; doi:10.1186/1750-2187-8-10)
Supplement: Additional file 1: Figure S1 — PKA activity assay in IGF1R-dependent GEO and CBS cells. Treatment with OSI-906 (1 μM) leads to increase in PKA activation. Forskolin was used as a positive control. Figure S2: MK-0646 activates PKA in FET Cells. Treatment with MK-0646, a humanized recombinant monoclonal antibody against IGF-1R activates PKA FET cells. Forskolin was used as a positive control. Figure S3: PARP activation by OSI-906 treatment is dependent on TGFβ/PKA signaling. Treatment with OSI906 (1 μM) for 4 h lead to PARP cleavage. Pretreatment with ALK5i (400nM) or H89 (10 μM) followed by OSI-906 treatment abrogate the PARP cleavage. Figure S4: TGFβ Signaling regulates AKAP 149/ PKARIIα interaction in FET cells. Treatment with TGFβ (5 ng/mL) for 4 h lead to robust increase in AKAP149/PKA RII interaction. Pretreatment with Ht31 (25 μM) followed by TGFβ treatment partially abrogated the AKAP149/PKA RII interaction. [file 1750-2187-8-10-S1.pdf]

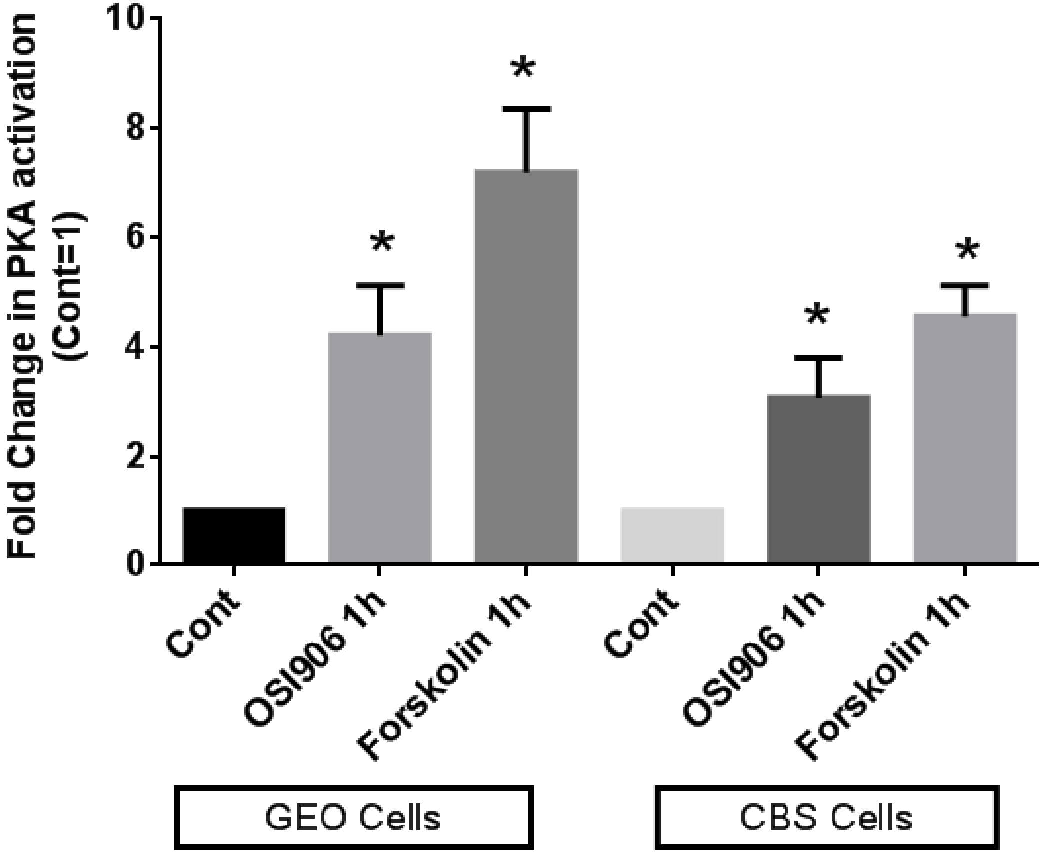

Supplemental Fig S1: PKA activity assay in IGF1R-dependent GEO and CBS cells. Treatment with OSI-906 (1µM) leads to increase in PKA activation. Forskolin was used as a positive control.

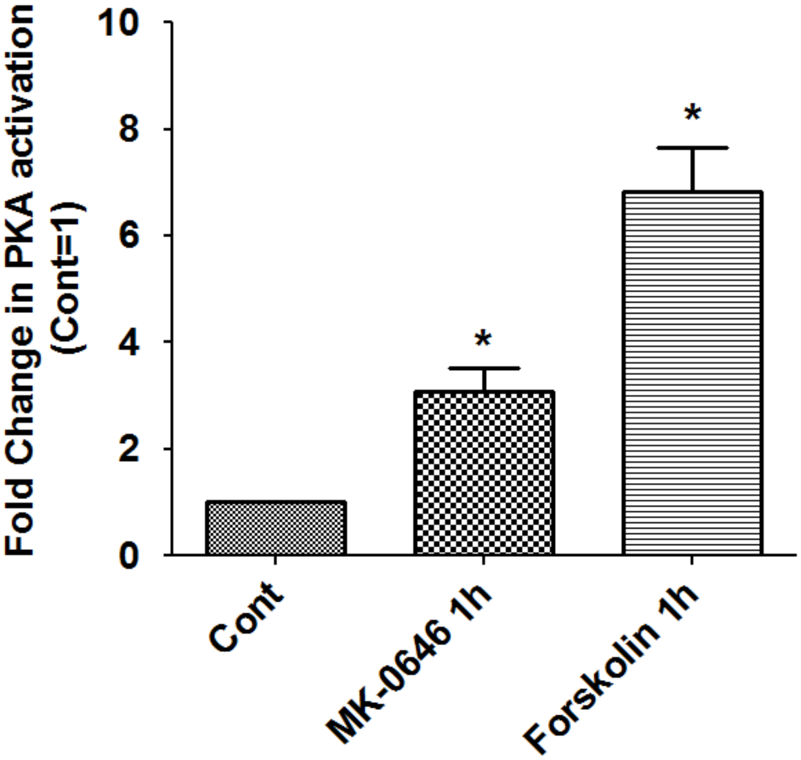

Supplemental Fig S2: MK-0646 activates PKA in FET Cells. Treatment with MK-0646, a humanized recombinant monoclonal antibody against IGF-1R activates PKA FET cells. Forskolin was used as a positive control.

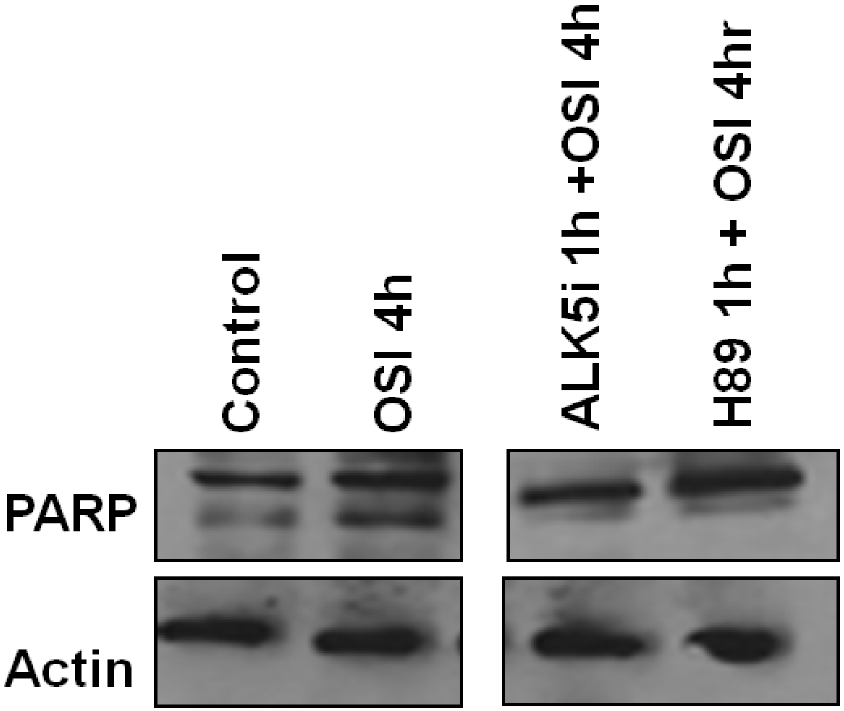

Supplemental Fig S3: PARP activation by OSI-906 treatment is dependent on TGFβ/PKA signaling. Treatment with OSI906 (1µM) for 4h lead to PARP cleavage. Pretreatment with ALK5i (400nM) or H89 (10µM) followed by OSI-906 treatment abrogate the PARP cleavage.

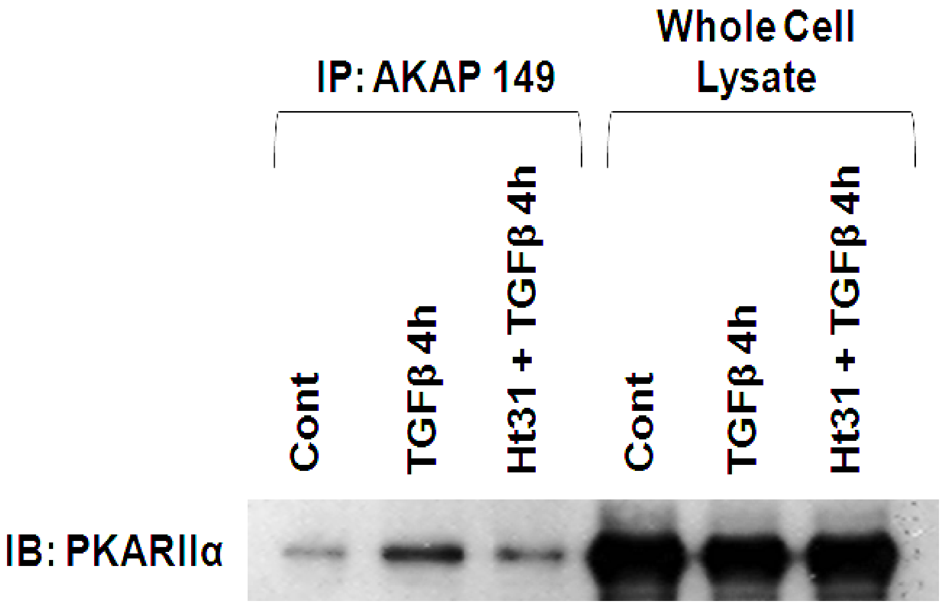

Supplemental Fig S4: TGFβ Signaling regulates AKAP 149/ PKARIIα interaction in FET cells. Treatment with TGFβ (5ng/mL) for 4h lead to robust increase in AKAP149/PKA RII interaction. Pretreatment with Ht31 (25μM) followed by TGFβ treatment partially abrogated the AKAP149/PKA RII interaction.
